# Bilateral Central Giant Cell Granuloma of the mandibular angle in three females from the same family

**DOI:** 10.1186/s13005-018-0171-7

**Published:** 2018-09-04

**Authors:** Simona Tecco, Silvia Caruso, Alessandro Nota, Pietro Leocata, Gianluca Cipollone, Roberto Gatto, Tommaso Cutilli

**Affiliations:** 1 Dental School, Vita-Salute San Raffaele University, via Olgettina 58, Milan, Italy; 2 School of Pediatric Dentistry, Department of Life, Health and Environmental Sciences, University of L’Aquila, Via Lorenzo Natali 1: Località Coppito, 67100 L’Aquila, Italy; 3 Pathology Unit, Post-graduated school of Pathology, Department of Life, Health & Environmental Sciences, University of L’Aquila, L’Aquila, Italy; 4 San Salvatore City Hospital L’Aquila, Unit of Pathology, Via Vetoio, 1, Coppito, 67100 L’ Aquila, AQ Italy; 5 Maxillo-Facial Surgery Unit, Department of Life, Health and Environmental Sciences, University of L’Aquila, Via Lorenzo Natali 1: Località Coppito, 67100 L’Aquila, AQ Italy

**Keywords:** Central Giant cells granuloma, Cherubism-like lesions, Case series

## Abstract

In literature there are few reports about multiple CGCG. But this is the first report of bilateral CGCG of the mandibular angles in three females from the same family.

This report describes three cases of females from the same family - a mother and two young daughters - with bilateral CGCG in their jaw angles. All the lesions were surgically removed and the histopathologic diagnosis was always identical: giant cell central granulomas, with patterns that were absolutely superimposable between them and with that of the mother.

The hypothesis is that this presentation of CGCG may be defined as hereditary bilateral CGCG of the mandibular angles (or also, cherubism-like lesions).

## Main text

Central giant cell granuloma (CGCG) is defined by the World Health Organization as an intraosseous lesion consisting of cellular fibrous tissue that contains multiple foci of haemorrhage, aggregations of multiple nucleated giant cells, and occasionally trabeculae of woven bone [1].

It is uncommon (7% of all benign jaw lesions), and the biologic behaviour ranges from quiescent to aggressive, with pain, root resorption and a tendency to recurrence after excision [1]. In the great part of cases, CGCG lesion is unilateral. Sometime the lesion is located in a mandibular angle. And very few rare cases are reported in literature of bilateral CGCG located at the two angles of the mandible [2–4].

A case of bilateral CGCG of the mandibular angle has been reported in a 12 years old female, and classified as idiopathic, as none of the family members of the young girl presented with a similar lesion [2]. Another sporadic case has been reported in an 18 years old girl, associated with neurofibromatosis type 1 [3]. Finally, another case of bilateral CGCG of the mandibular angle was reported in a 8 years old female with Noonan’s syndrome [4].

In this cases series, we describe the first report in literature of a repetitive bilateral CGCG of the two mandibular angles, in three females from the same family. These rare presentations of CGCG may be defined as hereditary bilateral CGCG of the mandibular angles or also cherubism-like lesions.

**Fig. 1:**
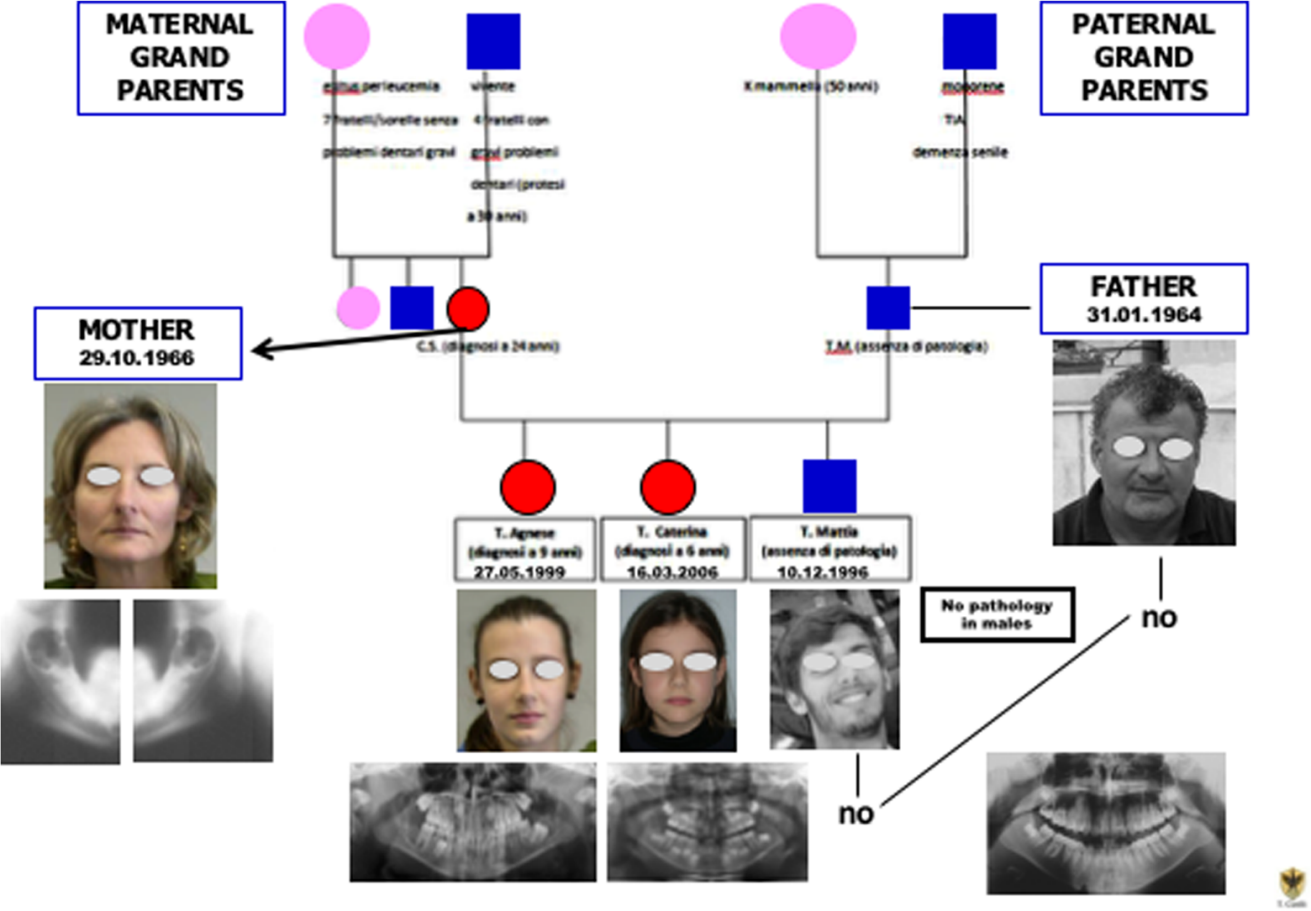
The family

In 1990, a 24-year-old young athlete was exposed to clinical observation at the maxillofacial surgery of the University of L’Aquila, central Italy, for the appearance of two osteolytic lesions at branches and mandibular angles (Fig. 1).

**Fig. 2:**
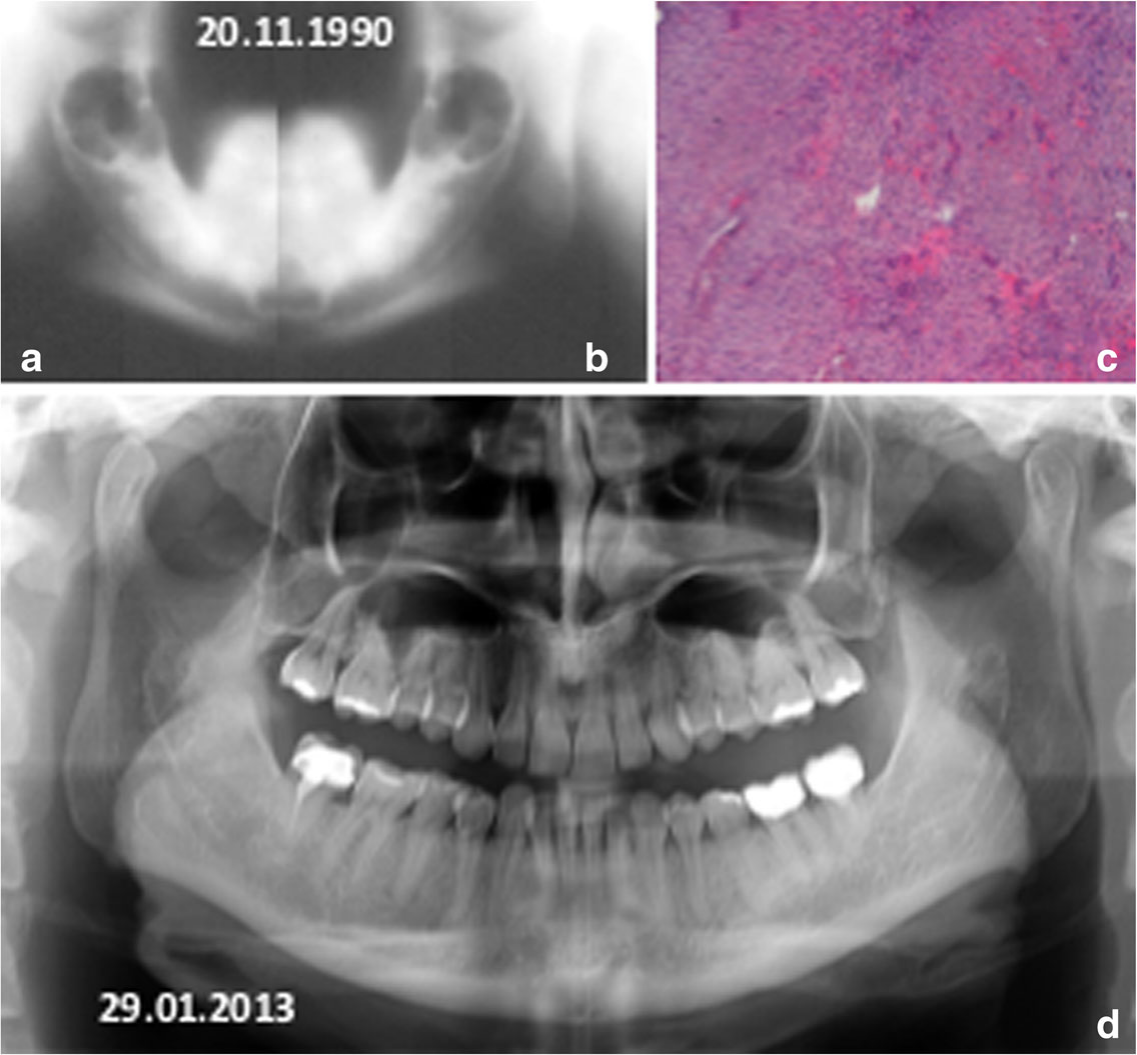
Mother: diagnosis at age 23. Mandibular x-Ray tomography: the right ramus (**a**) and the left ramus (**b**) show bilateral and symmetric radiolucenct areas. **c** Histopathological pattern suggests central giant cell granuloma. **d** Panoramic radiography 23 years after surgery; note the complete restoring of the mandibular bone structure

These lesions appeared symmetrical to radiological examinations (Fig. 2a, b).

The patient underwent surgical intervention and histological examination (Fig. 2c) revealed a case of GCGC. The patient was then subjected to regular follow-up over the years.

We currently have an x-ray performed after 23 years from surgery, which confirms the absence of relapses and a good mandibular bone restructuring.

After getting married in 1995 she had three children: a son in 1996 and two daughters, respectively, in 1999 and 2006.

The mother, due to her previous pathological lesion, had made radiological controls in childhood to the male child, with negative results.

**Fig. 3:**
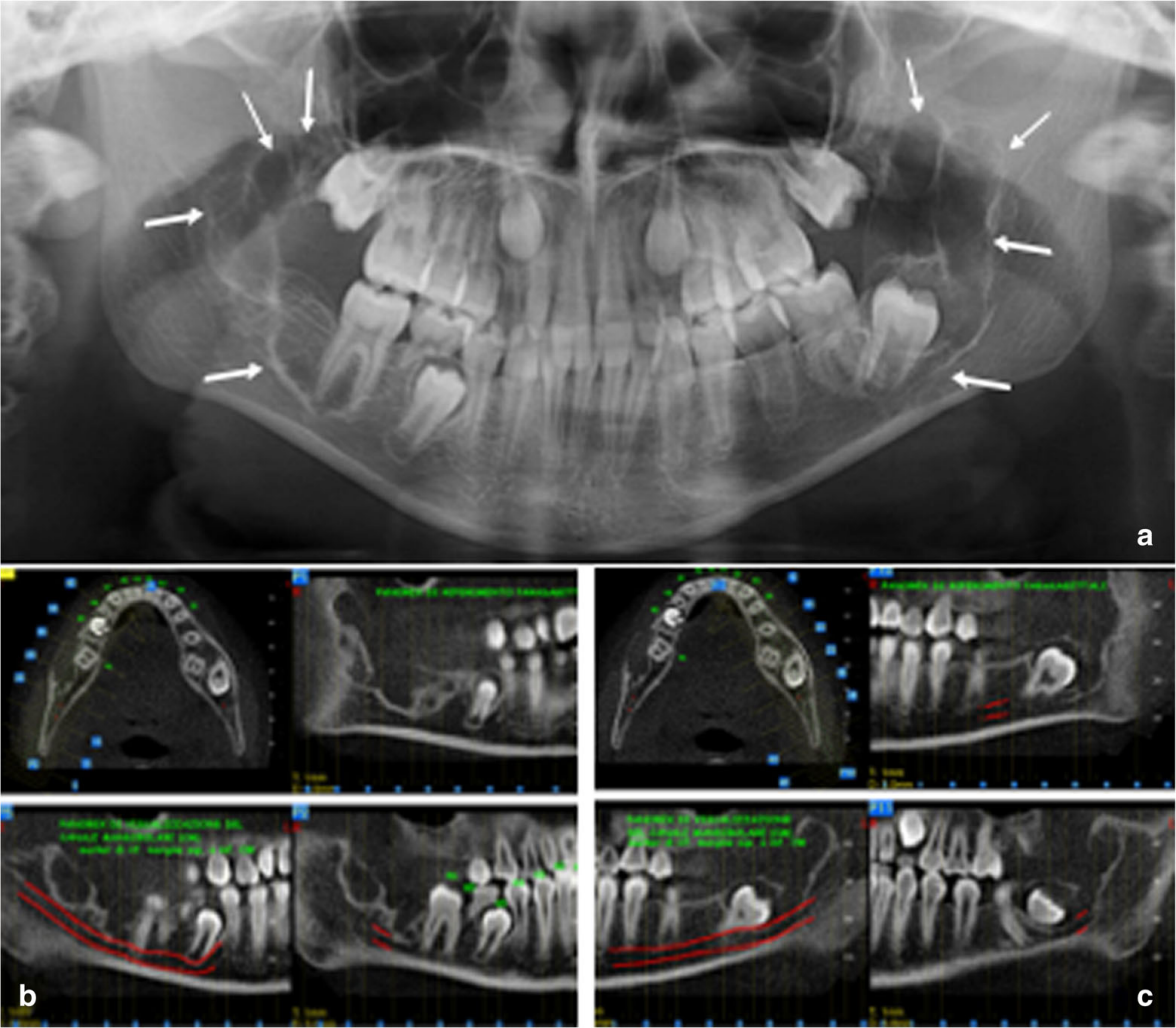
TA, female, diagnosis at age 9 *(4-gen-2008) -* **a** Panoramic radiography shows on both side of the mandible two symmetric large multilocular radiolucent lesions involving the angle and the ramus regions (white arrows). In the lower dental arch, there are only the first molar at right side (**b**) and the first and second molars at left side (**c**) CTCB study of the mandible, respectively, of the right and the left site, shows the extension of the lesions. Note their critical relationship with the mandibular canal and its neurovascular structures, in particular the inferior alveolar nerves

**Fig. 4:**
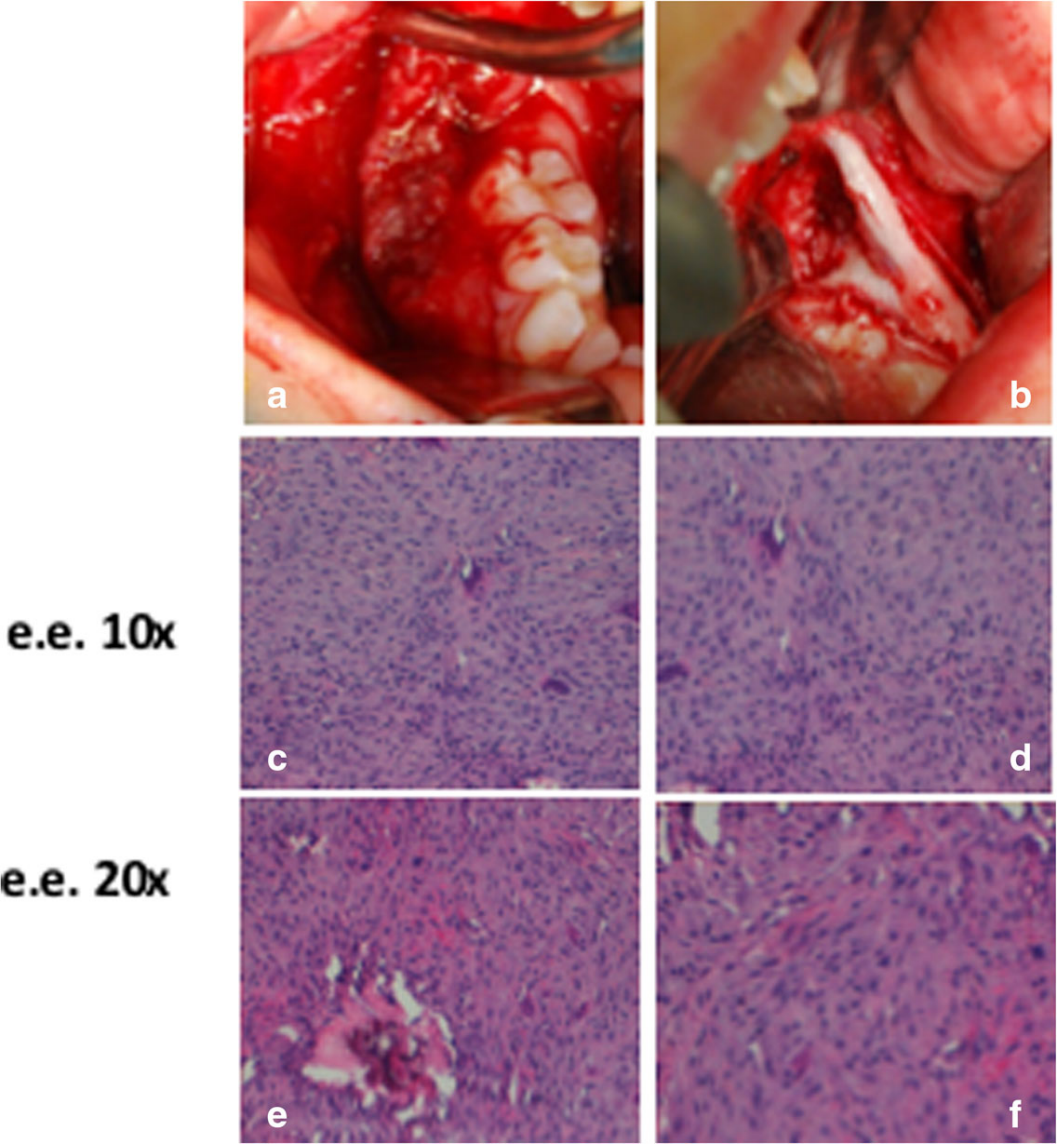
TA, female, diagnosis at age 9 – **a**-**c** and **d**-**f**: respectively the lesion of the right mandibular side and the left mandibular side. Note for each one the intraoperative aspect, and e.e. 10× and e.e. 20× histopatological speciments that show a moderately cellular and partially collagenized stroma, characterized by melted cells with dense nuclei and giant cells osteoclast like

On the contrary, at the age of 9 years, two symmetrical bilateral osteolytic lesions of the jaw were observed in the first female daughter, in the same sites as the mother (Figs. 3 and 4).

Subsequently, Cone beam CT scans showed the same lesions to the second female daughter, but earlier, at age of 6 years [5].

**Fig. 5:**
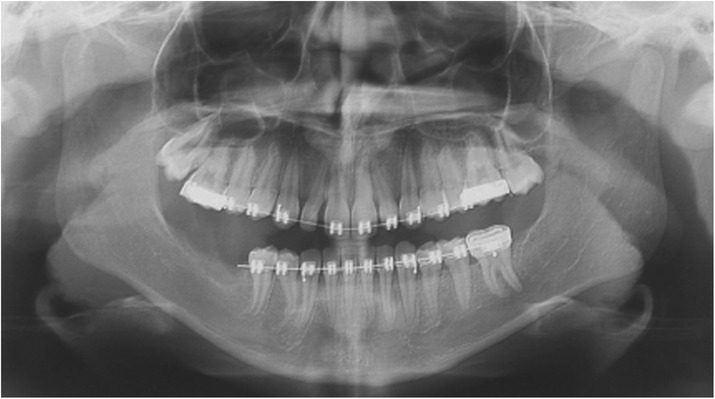
TA, female, diagnosis at age 9 – Follow-up at 5 years *(16-avr-2013)*. Panoramic radiography shows the good aspect of the bone mandibular structures and the absence of relapse

**Fig. 6:**
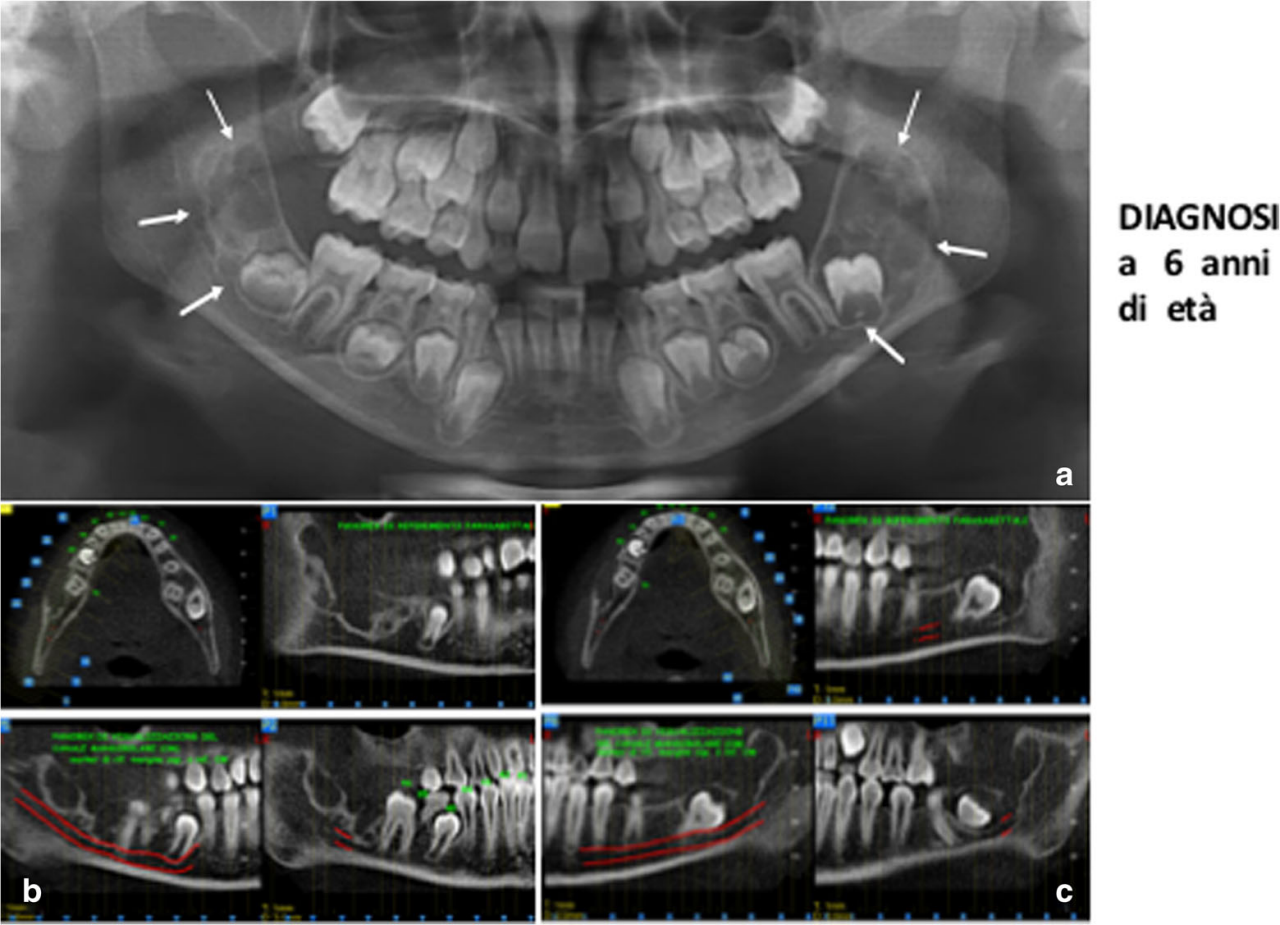
TC, female, diagnosis at age 6 (29-mar-2012) - **a** Panoramic radiography shows on both side of the mandible two symmetric large multilocular radiolucent lesions involving the angle and the ramus regions and the second molars. (white arrows). In the lower dental arch there are the first and the second molars (**b**, **c**) CTCB study of the mandible, respectively, of the right and the left site, shows the extension of the lesions. Note their critical relationship with the mandibular canal and its neurovascular structures, in particular the inferior alveolar nerves

**Fig. 7:**
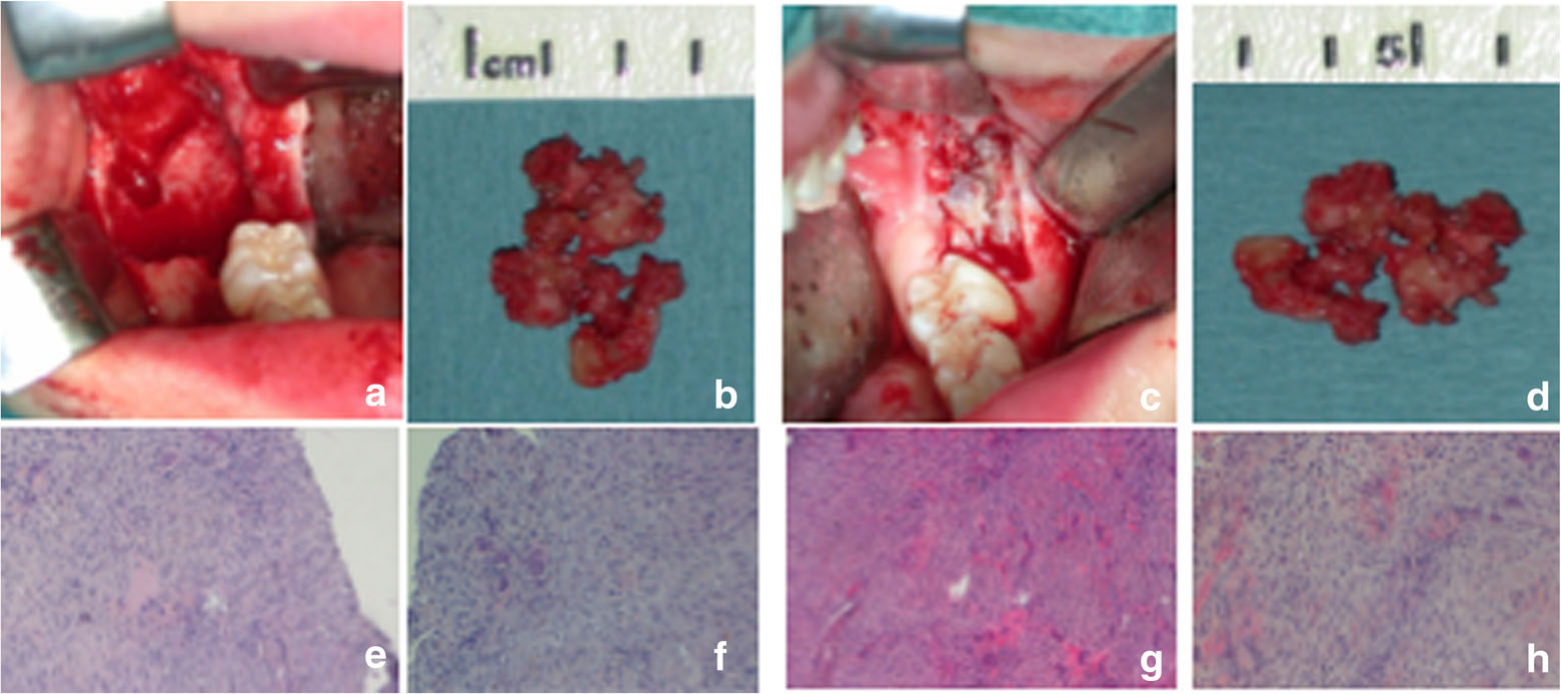
TA, female, diagnosis at age 9 – **a**-**d** and **e-h**: respectively the lesion of the right mandibular side and the left mandibular side. Note for each one the intraoperative aspect, the excised tissue, e.e. 10× and e.e. 20× histopatological speciments that show a moderately cellular and partially collagenized stroma, characterized by melted cells with dense nuclei and giant cells osteoclast like

**Fig. 8:**
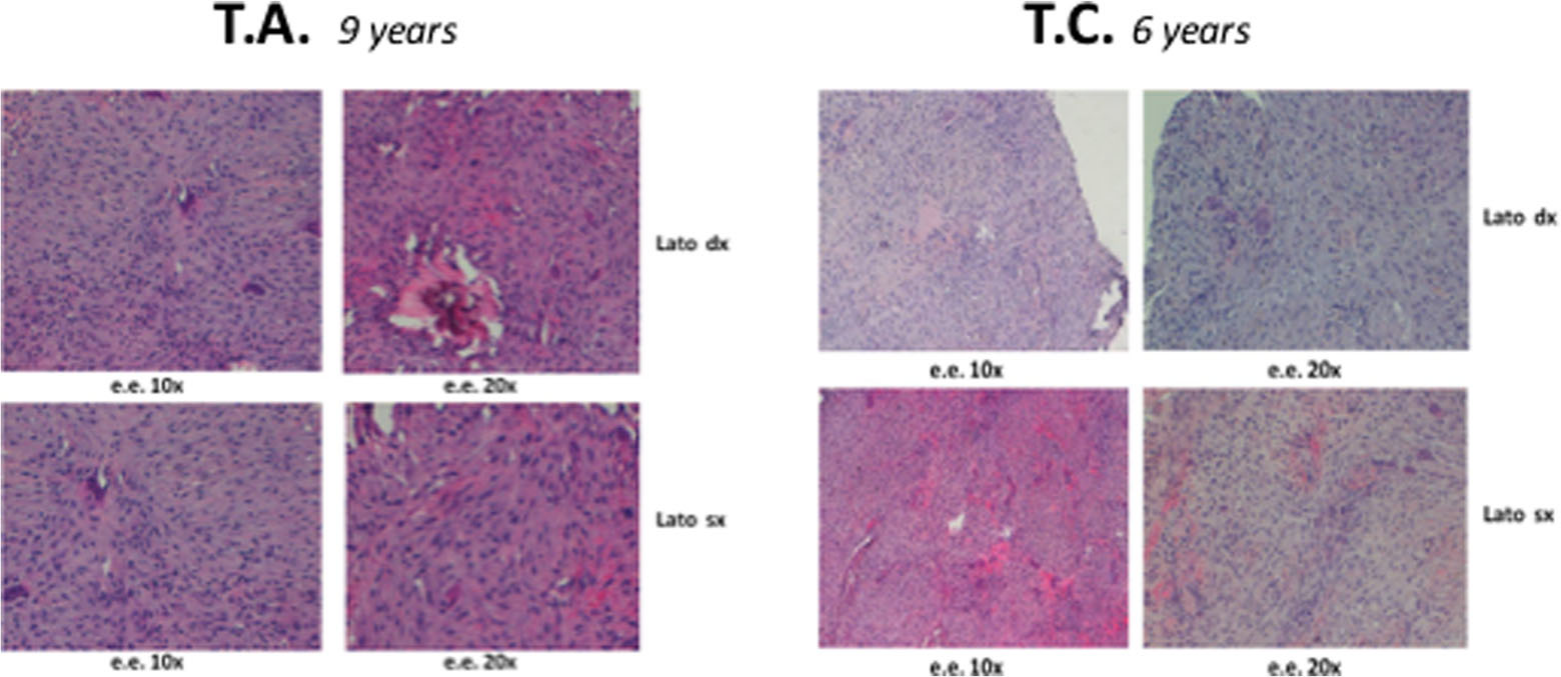
Absolute correspondence of the histological aspect of the lesions in the two sisters: the histological framework consisted of a moderately cellular and partially collagenized stroma, characterized by melted cells with dense nuclei and giant cells osteoclast like

All the lesions were surgically removed (Figs. 4a, 5, 6 and 7a, b, and f) and the histopathologic diagnosis was always identical (Fig. 8): giant cell central granulomas, with patterns that showed an absolute correspondence between them and with the mother (compare Figs. 2c,3, 4b, c, 5, 6 and 7c, d, g, h).

**Fig. 9:**
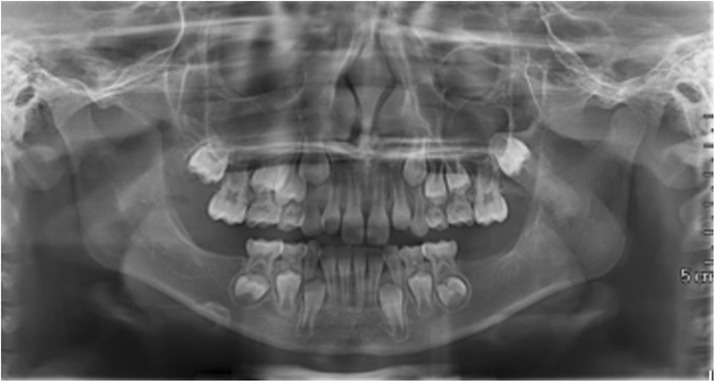
TA, female, diagnosis at age 9. Follow-up at 8 months years *(16-oct-2013)*. Panoramic radiography shows the good aspect of the bone mandibular structures and the absence of relapse

After the surgery, radiological follow-up examinations showed no relapses and good restructuring of the mandibular bone structure (Figs. 2c,3, 4, 5, 6, 7 and 9). The father was free from this disease. Periodical yearly follow-up was suggested for the two sisters until the end of puberty.

To the best of the authors’ knowledge, this is the first report of three cases of bilateral CGCG of the mandibular angles in three females from the same family. Considering the repetition of the lesion in subjects belonging to the same family, considering the particular location of the lesions (the mandibular angles in all three subjects), this situation may be attributed to the presence of a grade I (low level) Cherubism, or to the occurrence of cherubism-like lesions, as the cases did not show the other peculiar characteristics of Cherubism [6–8]. Table 1 shows the summary of the differences between Cherubism and idiopathic CGCG lesions that was followed in order to classify the lesion of the present cases. CGCG lesions may be associated with other disorders like Neurofibromatosis type 1 [3], gingival fibromatosis as well as Noonan’s syndrome [4], all of them are Rasopathies. Noonan syndrome is an autosomal dominantly inherited syndrome with variable expressivity. And multiple CGCG lesions in Noonan’s syndrome may be aggressive and cause complications. For these reasons, the diagnosis of Noonan’s syndrome was firstly taken in consideration. But the physical examination of these subjects contributed to discard the diagnosis of Noonan’s syndrome, that is characterized by short stature and atypical face like a broad or webbed neck, low set and posteriorly angulated ears, ptosis, hypertelorism, and downward-slanting eyes [9].

**Table 1:** Comparison of the characteristics of Cherubism and idiopathic CGCGs

	Cherubism	Idiopathic CGCG
Aetiology	Caused by a gain-of-function mutation in the gene coding a c-Abltyrosine kinase-binding protein (SH3BP2) located on the short arm of chromosome 4	The true aetiology is unknown and still controversial. It was thought that it is a reparative component. However, the evidence is not available to classify the lesions as reparative. The CGCG is thought by many to be reactive, but it is classified as a benign, non-neoplastic lesion.
Gender distribution	More diffused in males (or equally diffused between males and females)	More diffused in females
Age distribution	More prevalently diagnosed in children	CGCGs mainly affect patients between 10 and 30 years
Facial aspect	Swelling of bilateral mandibular angle region, typical of Cherubism (accompanied by hypertelorism)	Normal
Other signs	A marked cervical lymphadenopathy is common.	None
Definition (concept)	Cherubism is an autosomal dominantly inherited condition, with variable expressivity, that is characterized by multi-quadrant radiolucent lesions of the jaws and a progressive and clinically, symmetrical enlargement of the mandible and/or the maxilla.	Central giant cell granuloma (CGCG) is defined by the World Health Organization as an intraosseous lesion. The biologic behaviour ranges from quiescent to aggressive, with pain, root resorption and a tendency to recurrence after puberty.
Mandibular Lesions	Symmetrical mandibular lesions	Lesions are typically found unilaterally in the frontal region of the mandible. Sometime the lesion is located in a mandibular angle.
Family occurrence	There is usually a familial history of similarly affected family members.	Sometime they show an autosomal inheritance. In these cases, when bilateral, they are defined cherubism-like lesions.
Histological aspect of lesions	The lesions appear microscopically generally indistinguishable from CGCG, except occasionally, when a fairly characteristic condensation of perivascular collagen is evident	Cellular fibrous tissue that contains multiple foci of haemorrhage, aggregations of multiple nucleated giant cells, and occasionally trabeculae of woven bone.
Images and Rx aspects	Multi-quadrant radiolucent lesions of the jaws At the Rx can be observed a marked displacement or agenesia of second and third molars as well as premature exfoliation of primary teeth.	Osteolytic lesions of the jaw
Differential diagnosis	Neurofibromatosis type 1, gingival fibromatosis as well as Noonan’s syndrome, all of them are Rasopathies	Neurofibromatosis type 1, gingival fibromatosis as well as Noonan’s syndrome, all of them are Rasopathies
Treatments	Treatment of lesions consists of local curettage, jaw contouring, intralesional steroid injections, and systemic calcitonin administration as well	Commonly treated by surgical curettage.
Long-term clinical management	Long-term follow-up	Long-term follow-up
Prognosis	The regression of the lesions is often seen following puberty	These lesions tend to increase before the puberty (perhaps due to ovarian hormones) and to stabilize after puberty.

Cherubism is an autosomal dominantly inherited condition, with variable expressivity, that is characterized by multi-quadrant radiolucent lesions of the jaws and a progressive and clinically, symmetrical enlargement of the mandible and/or the maxilla [10–12]. There is usually a familial history of similarly affected family members and the regression of the lesions is often seen following puberty [8]. In the present family the mother was 24-year-old at the time of the first diagnosis, consequently she could probably be considered as a missed diagnosis until that age.

From a cellular point of view, the cherubism-like lesions appear microscopically generally indistinguishable from CGCG, except occasionally, when a fairly characteristic condensation of perivascular collagen is evident [10]. Consequently, the clinical aspects provide helpful clues to distinguish cherubism from CGCGs. CGCGs mainly affect patients between 10 and 30 years (while cherubism is more prevalent in children) and are typically found unilaterally in the frontal region of the mandible, whereas symmetrical lesions are found in cherubism [13]. The present cases show cherubism-like lesions.

Cherubism originates from genetic alteration in the SH3BP2 gene, and currently, it is believed to be caused by a gain-of-function mutation in the gene coding a c-Abltyrosine kinase-binding protein (SH3BP2) located on the short arm of chromosome 4 [14]. Only a sporadic case of CGCG with mutation of this gene was previously published [11, 15]. While another study conducted on a group of patients with an aggressive CGCG did not show any mutations, indicating that Cherubism is indeed a distinct entity from CGCG [16]. In the present cases, the patients do not present the typical swelling of bilateral mandibular angle region, typical of Cherubism (accompanied by hypertelorism^1^). But the repetition of the same cherubin-like lesions in three female subjects belonging to the same family, is suggestive for this diagnosis. Unfortunately, the family refused to perform genetic analysis to investigate the mutation in the SH3BP2 gene.

Dental findings in Cherubism include marked displacement of developing or agenesia of second and third molars as well as premature exfoliation of primary teeth [17]. In addition, in Cherubism a marked cervical lymphadenopathy is common.

In the present cases, there were not all common clinical aspects of Cherubism and only females were characterized by lesions. While Cherubism, in the scientific literature, is reported to be more common in males or equally distributed between males and females [17]. For the CGCG lesions, instead, the predominant distribution among females, respect to males, is certain [2], correlated to the hormonal influence due to ovarian hormones, oestrogen and progesterone, which are supposed to be responsible for the development of CGCG as for other pathologies [18–21].

For example, some cases of central giant cell lesion in pregnant patients have showed a proliferation, and also in subjects during a hormonal therapy [18]. But an immunostaining research, aimed to the detection of of estrogen and progesterone receptor proteins in 10 CGCG lesions, failed to evidence estrogen receptor protein, except for an occasional mononuclear cell stained weakly positive for estrogen receptor protein [18]. In other cases, estrogen receptor positivity was found in stromal cells. In ten of these, osteoclast-type giant cells also exhibited estrogen receptor immunostaining [22]. Due to the different results in literature, the direct influence of the ovarian hormones, estrogen and progesterone, in the development and growth of these lesions is still to be considered only a hypothesis.

For the present three cases, therefore, the hypothesis may be a hereditary form of bilateral CGCG of the mandibular angles - lesions that could be defined as cherubism-like lesions - or a rare manifestation of grade I Cherubism. CGCGs of the jaws are commonly treated by surgical curettage. And the management generally involves long-term follow-up, with the assumption that these lesions will stabilize during puberty. Thus, a yearly follow-up was suggested to the patients until the end of puberty.

## Conclusion

Three females from the same family presented identical bilateral CGCG of the mandibular angles. In literature there are few reports about multiple CGCG, but this case clearly report the autosomal inheritance of this pathology, and even with a repetitive cherubism-like location of the lesion at the mandibular angles. Thus, should be important to perform the genetic analysis in order to investigate the presence of the related gene mutations.

## Abbreviations

CGCG: Central Giant Cells Granuloma

## Footnotes

1 Clinically, cherubism most commonly manifests as a progressive and symmetrical enlargement of the mandible and/or the maxilla, and is first noted between between 2 and 7 years of age, after which, lesions proliferate and increase in size until puberty. The lesions subsequently begin to regress, fill with bone and remodel until age 30, when they are frequently not detectable. Mandibular swelling produces plump cheeks and maxillary enlargement causes retraction of the lower eyelids and elevation of the pupils upward, resulting in an “angel-like” appearance reminiscent of the cherubs depicted in Renaissance art.

## References

[1] Barnes L, Eveson JW, Reichart P (2005). Sidransky D (eds.): World Health Organization classification of tumors. Pathology genetics of head and neck tumors.

[2] Orhan E, Erol S, Deren O, Sevin A, Ekici O, Erdoğan B (2010). Idiopathic bilateral central giant cell reparative granuloma of jaws: a case report and literature review. Int J Pediatr Otorhinolaryngol.

[3] Chrcanovic BR, Gomez RS, Freire-Maia B (2011). Neurofibromatosis type 1 associated with bilateral central giant cell granuloma of the mandible. J Craniomaxillofac Surg.

[4] Edwards PC, Fox J, Fantasia JE, Goldberg J, Kelsch RD (2005). Bilateral central giant cell granulomas of the mandible in an 8-year-old girl with Noonan syndrome (Noonan-like/multiple giant cell lesion syndrome). Oral Surg Oral Med Oral Pathol Oral Radiol Endod.

[5] Saccucci M, D'Attilio M, Rodolfino D, Festa F, Polimeni A, Tecco S (2012). Condylar volume and condylar area in class I, class II and class III young adult subjects. Head Face Med.

[6] Jones WA, Gerrie J, Pritchard J (1950). Cherubism--familial fibrous dysplasia of the jaws. J Bone Joint Surg Br.

[7] Jones WA (1938). Further observations regarding familial Multilocular cystic disease of the jaws. Br J Radiol.

[8] Von Wowern N (2000). Cherubism: a 36-year long-term follow-up of 2 generations in different families and review of the literature. Oral Surg Oral Med Oral Pathol Oral Radiol Endod.

[9] Noonan JA (1994). Noonan syndrome. An update and review for the primary pediatrician. Clin Pediatr (Phila).

[10] Regezi JA (2002). Odontogenic cysts, odontogenic tumors, fibroosseous, and giant cell lesions of the jaws. Mod Pathol.

[11] Hyckel P, Berndt A, Schleier P, Clement JH, Beensen V, Peters H (2005). Cherubism - new hypotheses on pathogenesis and therapeutic consequences. J Craniomaxillofac Surg.

[12] Martínez-Tello FJ, Manjón-Luengo P, Martin-Pérez M, Montes-Moreno S (2005). Cherubism associated with neurofibromatosis type 1, and multiple osteolytic lesions of both femurs: a previously undescribed association of findings. Skelet Radiol.

[13] Kaugars GE, Niamtu J, Svirsky JA (1992). Cherubism: diagnosis, treatment, and comparison with central giant cell granulomas and giant cell tumors. Oral Surg Oral Med Oral Pathol.

[14] Ueki Y, Tiziani V, Santanna C, Fukai N, Maulik C, Garfinkle J (2001). Mutations in the gene encoding c-Abl-binding protein SH3BP2 cause cherubism. Nat Genet.

[15] Carvalho VM, Perdigão PF, Amaral FR, de Souza PEA, De Marco L, Gomez RS (2009). Novel mutations in the SH3BP2 gene associated with sporadic central giant cell lesions and cherubism. Oral Dis.

[16] de Lange J, van Rijn RR, van den Berg H, van den Akker HP (2009). Regression of central giant cell granuloma by a combination of imatinib and interferon: a case report. Br J Oral Maxillofac Surg.

[17] Papadaki ME, Lietman SA, Levine MA, Olsen BR, Kaban LB, Reichenberger EJ (2012). Cherubism: best clinical practice. Orphanet J Rare Dis.

[18] Whitaker SB, Bouquot JE (1994). Estrogen and progesterone receptor status of central giant cell lesions of the jaws. Oral Surg Oral Med Oral Pathol.

[19] Saccucci M, Polimeni A, Festa F, Tecco S (2012). Do skeletal cephalometric characteristics correlate with condylar volume, surface and shape? A 3D analysis. Head Face Med.

[20] Tecco S, Farronato G, Salini V, Di Meo S, Filippi MR, Festa F, D'Attilio M (2005). Evaluation of cervical spine posture after functional therapy with FR-2: a longitudinal study. Cranio..

[21] Tecco S, Nota A, Caruso S, Primozic J, Marzo G, Baldini A, Gherlone EF. Temporomandibular clinical exploration in Italian adolescents. Cranio. 2017:1–8. 10.1080/08869634.2017.1391963. Epub ahead of print. 10.1080/08869634.2017.1391963 29072541

[22] Günhan M, Günhan O, Celasun B, Mutlu M, Bostanci H (1998). Estrogen and progesterone receptors in the peripheral giant cell granulomas of the oral cavity. J Oral Sci.

